# Editorial: Mitogen Activated Protein Kinases

**DOI:** 10.3389/fcell.2017.00080

**Published:** 2017-09-14

**Authors:** Ana Cuenda, José M. Lizcano, José Lozano

**Affiliations:** ^1^Department of Immunology and Oncology, Centro Nacional de Biotecnología (CSIC) Madrid, Spain; ^2^Department of Biochemistry and Molecular Biology, Institute of Neurosciences, Faculty of Medicina, Universitat Autonoma de Barcelona Bellaterra, Spain; ^3^Department of Molecular Biology and Biochemistry, Universidad de Málaga Málaga, Spain

**Keywords:** MAPK, ERK1/2, p38MAPK, Erk5, scaffolding proteins

Mitogen-activated protein kinase (MAPK) cascades are among the most intensively studied signal transduction systems. MAPK pathways are evolutionarily conserved in all eukaryotes and allow cells to respond to changes in the physical and chemical properties of the environment and to produce an appropriate response by altering many cellular functions including cell differentiation, cell death, proliferation, metabolism rate, or the interaction with other cells. Four subfamilies of MAPKs have been extensively characterized in mammalian cells: ERK1/2, JNKs, p38s and ERK5 (Schaeffer and Weber, 1999; Cuenda and Rousseau, 2007; Gaestel et al., 2009; Kyriakis and Avruch, 2012; Arthur and Ley, 2013). All MAPK cascades comprise several molecular intermediaries at sequential level, which become activated in response to a broad panel of intra- and extra-cellular stimuli. They are typically organized in a three-kinase architecture consisting of a MAPK, a MAPK activator (MEK, MKK, or MAPK kinase), and a MEK activator [MEK kinase (MEKK)]. Transmission of signals is normally achieved by sequential phosphorylation and activation of the components specific to a respective cascade (Schaeffer and Weber, 1999; Kyriakis and Avruch, 2012).

In the past decade, there has been a vast increase of new works using different approaches and technologies that have provided valuable insight into the spatiotemporal dynamics, the regulation and functions of MAPK pathways, as well as their therapeutic potential. Since MAPK research is a very dynamic field, our aim planning this topic was to generate an opportunity in which MAPK researchers could make public their latest discoveries and also review and revisit different aspects of this research area.

Several key issues in the MAPK field are discussed in this topic. One of them is how selectivity and efficiency of MAPK pathways is preserved, despite the apparent ability of their components to function in multiple pathways. Particularly, Casar and Crespo describe recent findings on the ERK1/2 scaffold proteins, which maintain pathway integrity and signaling efficiency. Scaffold proteins connect different MAPK pathway elements into multi-enzymatic complexes (Kyriakis and Avruch, 2012), which fine-tune signal amplitude and duration, and provide signal fidelity by isolating these complexes from external interferences. Also, scaffold proteins are spatial regulators of MAPK signals, and depending on the subcellular localization from which the activating signals arise, defined scaffolds determine which substrates are phosphorylated. In this respect, Gaestel describes how the MAPKs ERK1/2 and p38 signal further downstream by the activation of the so-called MAPK-activated protein kinases (MAPKAPKs). He summarizes recent findings regarding the molecular basis of signaling complexes between MAPKs and MAPKAPKs and describes the non-canonical activation of the ERK1/2 substrate RSKs by p38-MK2/3 in dendritic cells. In his mini-review Gaestel also discusses recent challenges arising from off target effects of the widely used RSK inhibitors SL0101 and BI-D1870.

Functional redundancy between MAPKs is very common since there are more than one isoform at each level of the MAPK cascades. This issue is also discusses in the topic. Buscà et al. and Saba-El-Leil et al. focus their attention on ERK1 and ERK2. Buscà et al. collect data on ERK1 vs. ERK2 gene structures, protein sequences, expression levels, structural and molecular mechanisms of activation and substrate recognition, and very nicely perform a rigorous analysis of studies regarding the individual roles of ERK1 and ERK2. They conclude that ERK1 and ERK2 exhibit functional redundancy and propose the concept of the global ERK quantity as being the essential determinant to achieve ERK function. Saba-El-Leil et al. also point out evidence supporting the ERK1 and ERK2 redundant roles in embryonic development and in physiology, and in addition discuss the redundancy of JNK (JNK1/2/3) and p38 (p38α/β/γ/δ) isoforms.

Additionally, this topic includes some latest advances on MAPK function and implication in differentiation, inflammation and cancer. Two reviews focus on p38MAPK signaling in cell differentiation; particularly, Segalés et al. nicely summarize the molecular mechanisms implicated in the transition of muscle satellite cells throughout the distinct myogenic stages and also discuss recent findings on the causes underlying satellite cell functional decline with aging. They describe the important function of p38 in myogenesis, and in building up satellite cell adaptive responses in muscle regeneration; and discuss how these responses are altered in aging. On the other hand, Rodríguez-Carballo et al., discuss the role of MAPKs—centring on p38—on the regulation of transcription factors that are essential for adipocyte, chondrocytes, osteoblasts and osteoclasts differentiation and function. They also describe how inflammatory cytokines activate MAPKs during the differentiation process. It is well established that MAPKs are not only activated in response to inflammatory cytokines, but also serve as key regulators of pro-inflammatory cytokines biosynthesis, which makes different components of these pathways potential targets for the treatment of autoimmune and inflammatory diseases (Cuenda and Rousseau, 2007; Gaestel et al., 2009; Arthur and Ley, 2013). Lloberas et al., describe how the MAPK phosphatase MKP-1 is regulated, and also explain the balancing role of MKP-1 in the control of macrophage behavior by dephosphorylating MAPKs, which in turn have a strong impact in the inflammatory response since macrophages represent the primary host response to pathogen infection and link the immediate innate defense to the adaptive immune system. Reyskens and Arthur, review the last findings on MSK1/2, which are common p38 and ERK1/2 substrates. MSK1/2 are nuclear proteins that phosphorylate multiple substrates, including CREB or Histone H3, and are highly expressed in immune and nervous systems. The anti-inflammatory role of MSKs, by regulating the production of IL-10, and their implication in neuronal proliferation and synaptic plasticity in the central nervous system are described in this review. In addition, Richter et al. present their last data on the analysis of protein kinases during macrophage differentiation by using kinomics and phosphoproteomics in the human monocytic cell line THP-1. They find that monocyte-to-macrophage differentiation is associated with major rewiring of MAPK signaling networks and demonstrate that protein kinase MAP3K7 (TAK1) is critical for bacterial killing, chemokine production and differentiation.

Other process in which MAPKs are central elements is cancer development (Wagner and Nebreda, 2009; Dorard et al., 2017). Rousseau and Martel report an analysis of non-synonymous somatic mutations found in the TLR signaling network in lymphoid neoplasms. Lymphoid neoplasms form a family of cancers affecting B-cells, T-cells, and NK cells. The authors' findings suggest that TLR-mediated ERK1/2 activation via TPL2 is a novel path to tumorigenesis, and they propose that inhibition of ERK1/2 activation would prevent tumor growth in hematologic malignancies such as Waldenstrom's Macroglobulinemia, where the majority of the cells carry the MYD88[L265P] mutation. In the skin cancer context, Wellbrock and Arozarena review the complexity of the ERK signaling pathway in melanocytes, the healthy pigment cells that give rise to melanoma. They also discuss the mechanisms of action of different ERK-pathway inhibitors and their correlation with clinical response, the mechanisms of drug-resistance that limit patient's response, and new therapeutic opportunities for melanoma treatment targeting the ERK pathway. During the last decade members of the p38 signaling pathway have joined the group of canonical signaling pathways involved in tumor development and therefore are potential target for cancer treatment (Cuenda and Rousseau, 2007; Wagner and Nebreda, 2009). To this respect, García-Cano et al. summarize the role of p38MAPK in chemotherapy as well as the advantages that p38MAPK inhibition can bring to cancer therapy. The authors conclude that targeting p38MAPK for cancer treatment could be a double-edged sword depending on the patient's pathology and treatment.

Finally, two contributions, which shape the final outcome of the topic, address different aspects the some of the less studied MAPKs: ERK5, p38γ and p38δ. Gomez et al., review the role of ERK5 in regulating cell proliferation by mechanisms that are both dependent and independent of its kinase activity. They summarize the last findings regarding the complex regulation of ERK5 by upstream kinases and stabilizing chaperones in normal and cancer cells, and also during cell cycle. The authors describe the different mechanisms involved in the nuclear translocation of ERK5, -where mediates gene transcription- and discuss the possibility of targeting ERK5 to tackle different types of cancer. Escós et al. give a general overview of the recent advances made in defining the functions of the alternative p38, p38γ and p38δ, focusing in innate immunity and inflammation. They also discuss the potential of the pharmacological targeting of p38γ and p38δ pathways to treat autoimmune and inflammatory diseases, as well as cancer linked to inflammation.

We hope that all the information compiled in this eBook will be useful to researchers in this exciting field, and stimulate them to continue in their efforts to increase our knowledge on MAPK cascades. We want to acknowledge the great work of the authors, co-authors, and reviewers, and to thanks the superb support received from Frontiers Team members at all times.

## Author contributions

All authors listed have made a substantial, direct and intellectual contribution to the work, and approved it for publication.

### Conflict of interest statement

The authors declare that the research was conducted in the absence of any commercial or financial relationships that could be construed as a potential conflict of interest.
